# Red nucleus IL-33 facilitates the early development of mononeuropathic pain in male rats by inducing TNF-α through activating ERK, p38 MAPK, and JAK2/STAT3

**DOI:** 10.1186/s12974-021-02198-9

**Published:** 2021-07-05

**Authors:** Hao-Nan Li, Qing-Qing Yang, Wen-Tao Wang, Xue Tian, Fan Feng, Shu-Ting Zhang, Yu-Tong Xia, Jia-Xue Wang, Yuan-Wu Zou, Jun-Yang Wang, Xiao-Yan Zeng

**Affiliations:** 1 Department of Laboratory Medicine, The First Affiliated Hospital of Xi’an Jiaotong University, Xi’an, 710061 Shaanxi China; 2 Department of Pathogenic Biology and Immunology, Xi’an Jiaotong University Health Science Center, Xi’an, 710061 Shaanxi China; 3 Key Laboratory of Environment and Genes Related to Diseases (Xi’an Jiaotong University), Ministry of Education of China, Xi’an, China; 4 Biological Science BSc, Department of Biological Sciences, Xi’an Jiaotong-Liverpool University, Suzhou, 215123 Jiangsu China; 5 Biochemistry BSc, Faculty of Health and Life Sciences, University of Liverpool, Liverpool, L69 3BX UK

**Keywords:** Interleukin-33, Mononeuropathic pain, Red nucleus, Signaling pathway, Tumor necrosis factor-α

## Abstract

**Background:**

Our recent studies have identified that the red nucleus (RN) dual-directionally modulates the development and maintenance of mononeuropathic pain through secreting proinflammatory and anti-inflammatory cytokines. Here, we further explored the action of red nucleus IL-33 in the early development of mononeuropathic pain.

**Methods:**

In this study, male rats with spared nerve injury (SNI) were used as mononeuropathic pain model. Immunohistochemistry, Western blotting, and behavioral testing were used to assess the expressions, cellular distributions, and actions of red nucleus IL-33 and its related downstream signaling molecules.

**Results:**

IL-33 and its receptor ST2 were constitutively expressed in the RN in naive rats. After SNI, both IL-33 and ST2 were upregulated significantly at 3 days and peaked at 1 week post-injury, especially in RN neurons, oligodendrocytes, and microglia. Blockade of red nucleus IL-33 with anti-IL-33 neutralizing antibody attenuated SNI-induced mononeuropathic pain, while intrarubral administration of exogenous IL-33 evoked mechanical hypersensitivity in naive rats. Red nucleus IL-33 generated an algesic effect in the early development of SNI-induced mononeuropathic pain through activating NF-κB, ERK, p38 MAPK, and JAK2/STAT3, suppression of NF-κB, ERK, p38 MAPK, and JAK2/STAT3 with corresponding inhibitors markedly attenuated SNI-induced mononeuropathic pain or IL-33-evoked mechanical hypersensitivity in naive rats. Red nucleus IL-33 contributed to SNI-induced mononeuropathic pain by stimulating TNF-α expression, which could be abolished by administration of inhibitors against ERK, p38 MAPK, and JAK2/STAT3, but not NF-κB.

**Conclusions:**

These results suggest that red nucleus IL-33 facilitates the early development of mononeuropathic pain through activating NF-κB, ERK, p38 MAPK, and JAK2/STAT3. IL-33 mediates algesic effect partly by inducing TNF-α through activating ERK, p38 MAPK and JAK2/STAT3.

**Supplementary Information:**

The online version contains supplementary material available at 10.1186/s12974-021-02198-9.

## Introduction

Red nucleus (RN) is a prominent nucleus in extrapyramidal system, locating in the center of tegmental part of midbrain. It receives fiber projections mainly from cerebral cortex and cerebellum, and transmits to the spinal cord Rexed’s laminae V and VI as well as in the dorsal part of lamina VII through the rubrospinal tract [1]. Traditional wisdom holds that the major function of RN is to adjust muscle tension, coordinate movement, and attend sensorimotor integration [2–6]. However, increased evidence shows that the RN is also involved in sensory regulation. Functional magnetic resonance imaging studies indicate that the RN can be activated by painful stimuli, and the activity of RN is more driven by the requirements for sensory processing than by motor coordination per se [7, 8]. Morphological and physiological studies demonstrate that the RN receives a large amount of peripheral sensory information through indirect or direct pathways. Peripheral noxious stimuli change the electrical activity of RN neurons [9, 10], and electrical stimulation of the RN affects the spontaneous discharge and nociceptive reaction of neurons in the spinal dorsal horn and ventral posterolateral thalamic nucleus [11, 12]. Furthermore, behavioral experiments display that intrarubral administration of morphine markedly raises the pain threshold of thermal stimulation and produces an antinociception in naive rats [9].

Although previous researches have proven the involvement of RN in pain regulation, the underlying molecular mechanisms remain largely unclear so far. Our recent studies have shown that the RN modulates neuropathological pain through secreting cytokines. Following spared nerve injury (SNI) of rat sciatic nerve, proinflammatory cytokine tumor necrosis factor-α (TNF-α) is elevated in the RN at 1 week post-injury, and peaked at 2 weeks, while interleukin-1β (IL-1β) and IL-6 are respectively increased in the RN at 2 weeks and 3 weeks post-injury. TNF-α, IL-1β, and IL-6 all produce algesic effect in the development and/or maintenance of mononeuropathic pain [13–19]. Meanwhile, anti-inflammatory cytokine IL-10 is also upregulated in the RN at 3 weeks post-SNI, but exerts analgesic effect in SNI-induced mononeuropathic pain [20]. However, another anti-inflammatory cytokine-transforming growth factor-β (TGF-β) is downregulated in the RN at 1 week and reduced to the bottom level at 2 weeks post-SNI, although it produces antinociceptive effect under physiology condition [21]. These results clearly show that the RN contributes to the development and maintenance of chronic pathological pain, and it dual-directionally regulates mononeuropathic pain through secreting proinflammatory and anti-inflammatory cytokines.

Alarmin IL-33 is an important cytokine of the IL-1 superfamily; it widely exists in a variety of tissues and plays vital actions in tissue function and immune-mediated diseases. IL-33 is considered to be a dual-functional protein, regulating transcription intracellularly as a nuclear factor, and functioning extracellularly as a potent cytokine. IL-33 receptor is a heterodimer made up of ST2 and IL-1RAcP (IL-1 receptor accessory protein) [22–24]. Accumulated evidence shows that IL-33 is extensively distributed in the peripheral and central nervous system, and exerts critical actions in the pathogenesis of numerous nervous system diseases, such as trauma, infection, cerebrovascular diseases, and neurodegenerative diseases [25]. Recently, several researches have revealed that IL-33 contributes to nociceptive information processing and pain regulation too. Administration of exogenous IL-33 to normal mice evokes allodynia and hyperalgesia symptoms, while injection of IL-33 into *ST2*^−/−^ mice cannot induce pain-related behaviors [26–28]. Furthermore, upregulated IL-33 and ST2 are detected in the animal with pathological pain, blocking IL-33 with anti-IL-33 neutralizing antibody or knockout of ST2 gene relieves the symptoms of pathological pain [28–30]. Moreover, IL-33 has been proven to promote the development of cancer pain [31]. Based on the above findings, IL-33 is likely to partake in pain regulation and produces facilitatory effect at spinal cord and peripheral levels. However, it is unclear whether IL-33 in the RN is also involved in pain regulation. In the present study, we demonstrated that IL-33 and ST2 are constitutively expressed in the RN of male rats, and attend the regulation of mononeuropathic pain. IL-33 exerts an algesic effect in the early development stage of SNI-induced mononeuropathic pain, which relies on the activation of nuclear factor-κB (NF-κB), extracellular signal-regulated kinase (ERK), p38 mitogen-activated protein kinase (p38 MAPK), and Janus kinase 2/signal transducer and activator of transcription 3 (JAK2/STAT3). IL-33 facilitates the early development of mononeuropathic pain at least in part by inducing TNF-α through activating ERK, p38 MAPK and JAK2/STAT3.

## Materials and methods

### Animal

In view of the sex-dependent mechanism of mononeuropathic pain [32], only male Sprague-Dawley rats (200–230 g) provided by the animal experimental center of Xi’an Jiaotong University, China, were used in the present study. All rats were maintained on a 12:12 h light/dark cycle, and group-housed with water and food ad libitum. All animal studies were authorized by the Biomedical Ethics Committee of Xi'an Jiaotong University, and performed strictly following the ethical guidelines for the study of pain in animals [33].

### Spared nerve injury

Under the anesthesia of intraperitoneal injection of urethane (1.4 g/kg body weight), the right sciatic nerve and its three branches were carefully exposed and separated. The common peroneal nerve and the tibial nerve were ligated with 5-0 surgical silk thread and severed at the distal side, while the sural nerve should be kept intact. After this, the muscle and skin were then sutured layer by layer. The operation process of sham-surgery rats was the same as that of SNI rats, but the nerves were not ligated. The brain tissues were collected 1 week post-SNI or at the maximum effect time point of the test drugs.

### Intracerebral catheter implantation and drug administration

On the basis of the three-dimensional parameters of RN (5.2–6.7 mm behind bregma, 0.6–1.4 mm beside the midline, and 6.4–7.4 mm under the cortex) [34], a stainless steel catheter with a plug was vertically implanted into rat brain under the anesthesia of urethane (1.4 g/kg body weight), the end of the catheter was placed at 2.0 mm above the left RN.

After 1 week recovery, the plug was pulled out from the catheter and the test drug (0.5 μl) was administrated to the RN under the anesthesia of isoflurane (RWD Life Science Co, China) using a microsyringe with tip 2.0 mm beyond the end of the catheter. Recombinant rat IL-33 (10 ng/μl, 20 ng/μl, 40 ng/μl; Abcam, #ab200250) and rabbit anti-rat IL-33 neutralizing antibody (200 ng/μl, 500 ng/μl, 1000 ng/μl; ProSci, #4273) were made up with 0.9% saline. Inhibitors of JAK2 (AG490, 10 μg/μl; Abcam, #ab120950), ERK (PD98059, 5.0 μg/μl; Abcam, #ab120234), p38 MAPK (SB203580, 20 μg/μl; Abcam, #ab120162), and NF-κB (PDTC, 200 ng/μl; Sigma, #P8765) were first prepared with DMSO and then diluted with 0.9% saline to a final concentration of 10% DMSO. Doses of drugs were chosen according to the previous studies and proven to be effective [14, 19, 35]. Anti-IL-33 antibody and different signaling pathway inhibitors were administrated to the RN at 1 week post-SNI. IL-33 was administrated to the RN of naive rats directly or at 30 min after the pretreatment with different signaling pathway inhibitors. Then, 10% DMSO or 0.9% saline was used as vehicle control. After the experiments, toluidine blue (0.1%) staining was applied to confirm whether the drug was injected into the RN (Suppl. Fig. S1A).

### Mechanical hypersensitivity induced by IL-33

One week after intracerebral catheter implantation, 20 ng of recombinant rat IL-33 was injected into the left RN of naive rat to induce mechanical hypersensitivity since this dose could mimic the amount of IL-33 in the RN of SNI rats (1 week post-injury) (Suppl. Fig. S1C). IL-33 pre-absorbed by anti-IL-33 antibody or 0.9% saline alone was administrated as negative control. The brain tissues were collected at the maximum effect time point of IL-33.

### Behavioral testing

#### Mechanical paw withdrawal threshold measurement

Mechanical Dynamic Plantar Aesthesiometer (Ugo Basile, Italy) was applied to measure the paw withdrawal threshold (PWT) of rat. Before the behavioral experiment, rats were placed on the test arena for 30 min every day to familiar with the circumstance, and lasted at least 3 days. For formal measurement, the rat was singly put beneath a transparent plexiglass box on the metal mesh, and the sural innervation region was stimulated by a measuring steel needle (0.5 mm in diameter) with force ascending gradually from 0 to 50 g within 30 s. The monitor recorded the PWT automatically when the rat withdrew its hindpaw.

#### Footprint test

To explore whether intrarubral administration of anti-IL-33 antibody or IL-33 affects the locomotion of rats, a footprint test was conducted after injection of anti-IL-33 antibody into the RN of SNI rats or IL-33 into the RN of naive rats. The hindpaws of rats were dipped in black non-toxic paint and the rats were allowed to walk through a plastic tunnel (70 × 12 × 15 cm^3^, length × width × height), whose floor was covered with a sheet of white paper. Then, 5–6 steps were selected to analyze the step length and gait width as previously described [36].

### Immunohistochemical staining

Under anesthesia condition, rats were perfused via aortic intubation. At first, 200 ml normal saline was used for rapid flushing until the outflow was clear, and then 300 ml 4% paraformaldehyde (PFA) was slowly perfused for fixation. The brain tissue containing RN was took out and post-fixed in 4% PFA for 1 day, and then dehydrated with 30% sucrose and embedded into optimal cutting temperature compound. The coronal tissue slices (10 μm thick) were made by a cryostat microtome and used for the later histological studies.

After the usual proceedings, the sections were reacted with rabbit-derived anti-rat IL-33 antibody (1:400; ProSci, #4273), anti-rat ST2 antibody (1:500; Proteintech, #11920-1-AP), anti-rat NF-κB p65 antibody (1:200; Boster, Wuhan, China, #BA0610), anti-rat p-ERK1/2 antibody (1:50; CST, #4376), anti-rat p-JNK1/2/3 antibody (1:200; Abcam, #ab124956), anti-rat p-p38 MAPK antibody (1:50; CST, #4511), anti-rat p-JAK2 antibody (1:100; ImmunoWay, #YP0306), anti-rat p-STAT3 antibody (1:100; CST, #9145S), anti-rat p-AKT antibody (1:400; R&D, #AF887), or anti-rat TNF-α antibody (1:100; Abcam, #ab6671) overnight at 4 °C. Then the goat anti-rabbit IgG working solution (Boster, Wuhan, China, #SV0002) labeled with polymerized HRP was added and reacted 30 min at 37 °C. Finally, chromogenic agent 3,3′-diaminobenzidine tetrahydrochloride (Zsbio, Beijing, China) was used to develop the color. For control groups, the primary antibody was missed or supplanted by normal rabbit IgG. The histological images were taken using a Carl Zeiss microscope (Axio Scope A1), and the mean optical densities (MOD) of RN region were analyzed using ImageJ software (National Institute of Health, USA).

### Immunofluorescence staining

After blocking with normal goat serum, the sections were reacted with rabbit-derived anti-rat IL-33 antibody (1:400; ProSci, #4273) or anti-rat ST2 antibody (1:500; Proteintech, #11920-1-AP) mixed with mouse-derived anti-rat NeuN (1:1000; Abcam, #ab104224; marker of neuron), anti-rat OX42 (1:100; Millipore, #CBL1512; marker of microglia), anti-rat GFAP (1:250; Sigma, #G3893; marker of astrocyte), anti-rat O4 (1:250; Sigma, #O7139; marker of oligodendrocyte), or anti-rat CD31 (1:300; Santa Cruz, #sc-376764; marker of endothelial cell) overnight at 4 °C. Then the mixture of FITC-labeled goat anti-mouse IgG antibody (1:250; Abcam; green fluorescence) and Cy3-labeled goat anti-rabbit IgG antibody (1:2500; Abcam; red fluorescence) were added and reacted 1 h at 37 °C. Finally, the slides were counterstained with 4′,6-diamidino-2-phenylindole (DAPI) and observed on a Carl Zeiss microscope (Axio Scope A1) fluorescent microscope. To further verify the specificity of anti-IL-33 antibody and anti-ST2 antibody, brain slices from *IL33*^−/−^, *ST2*^−/−^, and wild-type (WT) male C57BL/6 mice (friendly provided by Prof. Fang Zheng, Department of Immunology, School of Basic Medicine, Tongji Medical College, Huazhong University of Science and Technology, China) were stained based on their reactivity with mouse IL-33 and ST2.

### Western blotting

The fresh RN tissue of rats was quickly removed under the anesthesia of urethane (1.4 g/kg body weight) (Suppl. Fig. S1B), and put into the refrigerant radio immunoprecipitation assay lysis buffer containing inhibitors against protease and phosphatase (Bimake, USA). After tissue homogenization and protein quantification, 20 μg/lane was loaded for electrophoresis and membrane transfer. The polyvinylidene difluoride membrane was blocked 2 h with 5% skim milk and reacted subsequently with rabbit-derived anti-rat IL-33 antibody (1:1000; ProSci, #4273), anti-rat ST2 antibody (1:1000, Proteintech, #11920-1-AP), anti-rat NF-κB p65 antibody (1:400, Boster, Wuhan, China, #BA0610), anti-rat p-ERK1/2 antibody (1:800, CST, #4376), anti-rat p-JNK1/2/3 antibody (1:3000, Abcam, #ab124956), anti-rat p-p38 MAPK antibody (1:300, Wanlei, Shenyang, China, #WL03428), anti-rat p-JAK2 antibody (1:1000; ImmunoWay, #YP0306), anti-rat p-STAT3 antibody (1:1000, CST, #9145S), anti-rat p-AKT antibody (1:800, R&D, #AF887), anti-rat TNF-α antibody (1:500, Boster, Wuhan, China, #PB0082), or anti-rat glyceraldehyde-3-phosphate dehydrogenase (GAPDH) antibody (1:5000, Proteintech, #10494-1-AP) overnight at 4 °C. Then HRP-labeled anti-rabbit IgG antibody (1:5000, Zsbio, Beijing, China, #ZB-5301) was added and reacted 1 h under room temperature. Finally, enhanced chemiluminescence solution (Boster, Wuhan, China) was used to develop the color, and Fusion FX5 camera system was applied for capturing photos. Images were analyzed using ImageJ software and normalized to GAPDH.

### Statistical analysis

Data were presented as the mean ± standard error (S.E.M.). The doses-effect relationship of drug was evaluated by Pearson correlation coefficient. The protein expression and locomotion difference among different groups were analyzed by one-way analysis of variance (ANOVA) with post hoc Bonferroni correction test. The effect difference of drug among different groups was analyzed by two-way repeated measures of ANOVA with post hoc Bonferroni correction test. *P* < 0.05 was considered the criteria of significance.

## Results

### Increased expressions of IL-33 and ST2 in the red nucleus of SNI rats

**Fig. 1:**
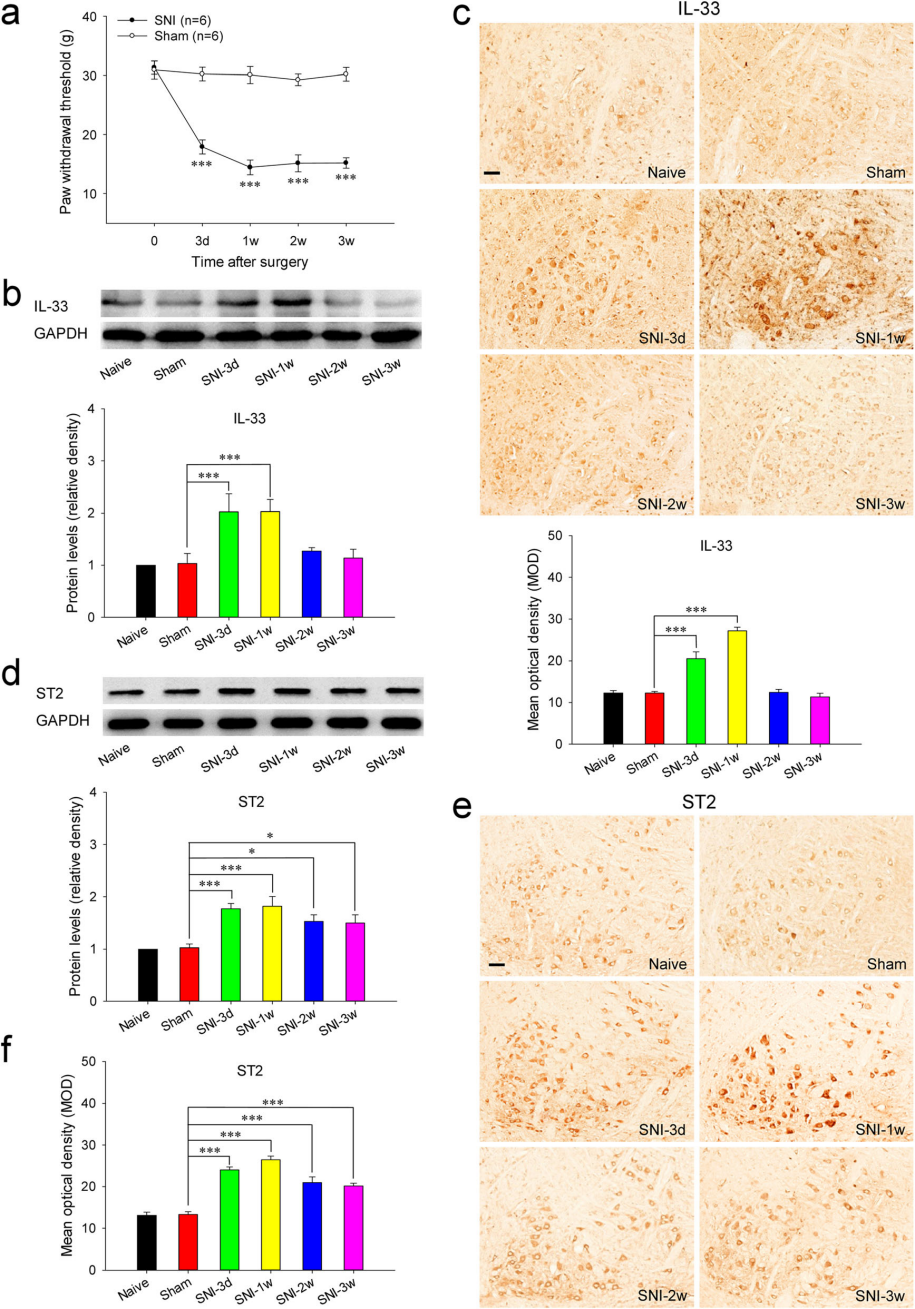
Increased expressions of IL-33 and ST2 in the RN of SNI rats. **A** Mononeuropathic pain induced by SNI (*n* = 6 per group, *F* = 198.886, *P* < 0.001). **B** Western blotting showed that red nucleus IL-33 was increased at 3 days, peaked at 1 week and returned to normal level at 2 weeks post-SNI (*n* = 6 per group, *F* = 9.435, *P* < 0.001). **C** Immunohistochemistry indicated that red nucleus IL-33 was upregulated at 3 days, peaked at 1 week and returned to normal level at 2 weeks post-SNI (*n* = 4 per group, *F* = 50.817, *P* < 0.001). **D** Western blotting showed that red nucleus ST2 was increased at 3 days, peaked at 1 week, and still remained at a high level at 3 weeks post-SNI (*n* = 6 per group, *F* = 7.693, *P* < 0.001). **E**, **F** Immunohistochemistry (*n* = 4 per group) indicated that red nucleus ST2 was upregulated at 3 days, peaked at 1 week, and still kept at a high level at 3 weeks post-SNI (*n* = 4 per group, *F* = 39.934, *P* < 0.001). *P < 0.05 and ***P < 0.001. Scale bars = 50 μm

After spared nerve injury, the mechanical PWT of injured hindpaw was significantly reduced at 3 days, and maintained at a low level 1 week later (Fig. 1A). During the whole experiment, no significant PWT changes were measured in the uninjured hindpaw of SNI rats (data not shown) and the hindpaw of sham-surgery rats compared to that before operation.

**Fig. 2:**
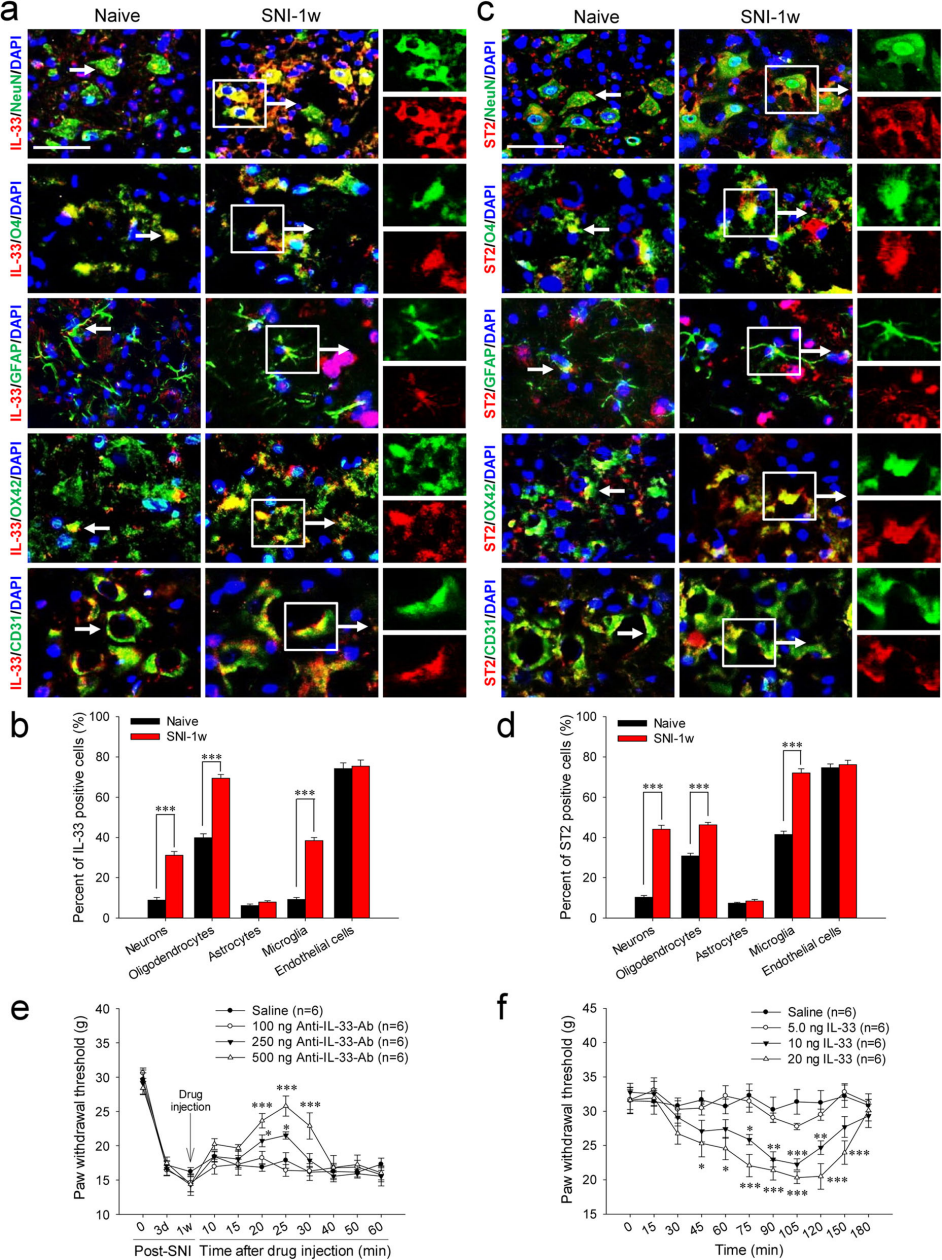
Cellular distributions of IL-33 and ST2 in the RN of SNI rats, and the effect of IL-33 in the early development of mononeuropathic pain. **A** Representative images of IL-33 (Red) co-localized with RN neurons, oligodendrocytes, astrocytes, microglia or endothelial cells (Green) in naive and SNI rats. **B** Semiquantitative analysis of IL-33-positive cells in the RN of naive and SNI rats (neurons: *F* = 104.519, *P* < 0.001; oligodendrocytes: *F* = 121.789, *P* < 0.001; microglia: *F* = 251.137, *P* < 0.001) (*n* = 4 per group). **C** Representative images of ST2 (Red) co-localized with RN neurons, oligodendrocytes, astrocytes, microglia, or endothelial cells (Green) in naive and SNI rats. **D** Semiquantitative analysis of ST2-positive cells in the RN of naive and SNI rats (neurons: *F* = 233.032, *P* < 0.001; oligodendrocytes: *F* = 76.744, *P* < 0.001; microglia: *F* = 155.490, *P* < 0.001) (*n* = 4 per group). **E** Intrarubral administration of anti-IL-33 antibody at 1 week post-SNI dose-dependently (*r* = 0.961, *P* = 0.0385) attenuated SNI-induced mononeuropathic pain compared to saline control (*n* = 6 per group, *F* = 7.166, *P* = 0.003). **F** Intrarubral administration of exogenous IL-33 to naive rats dose-dependently (*r* = − 0.955, *P* = 0.0454) evoked a mechanical hypersensitivity compared to saline control (*n* = 6 per group, *F* = 35.194, *P* < 0.001). *P < 0.05, **P < 0.01, and ***P < 0.001. Scale bars = 50 μm

In naive rats, IL-33 and ST2 were constitutively expressed in the RN (Fig. 1B–F). IL-33 was synthesized mainly in oligodendrocytes and endothelial cells, and a few in neurons, astrocytes and microglia (Fig. 2A, B). ST2 was mainly expressed in oligodendrocytes, microglia, and endothelial cells, together with a small amount in neurons and astrocytes (Fig. 2C, D). In SNI rats, red nucleus IL-33 and ST2 were significantly increased at 3 days post-injury, and peaked at 1 week compared to that in sham-surgery rats (Fig. 1B–F). Both IL-33 and ST2 were upregulated in neurons, oligodendrocytes, and microglia, but not astrocytes and endothelial cells (Fig. 2A–D). The protein expression of red nucleus IL-33 returned to normal level at 2 weeks post-SNI, while the expression of red nucleus ST2 remained at a high level at least until 3 weeks post-SNI (Fig. 1B–F). Additionally, no specific signals of IL-33 and ST2 were detected respectively in the RN of *IL-33*^−/−^ and *ST2*^−/−^ mice, indicating the specificity of anti-IL-33 antibody and anti-ST2 antibody we used (Suppl. Fig. S1D). These results imply that IL-33 probably attends the early development of mononeuropathic pain.

### Red nucleus IL-33 facilitates the early development of mononeuropathic pain

To assess the action of red nucleus IL-33 in the early development stage of SNI-induced mononeuropathic pain, we first observed the PWT changes in SNI rats after blocking red nucleus IL-33 with neutralizing antibody. As illustrated in Fig. 2E, intrarubral administration of various dosages of anti-IL-33 antibody (100 ng, 250 ng, 500 ng) at 1 week post-SNI raised the mechanical PWT of injured hindpaw (but not intact hindpaw) and attenuated SNI-induced mononeuropathic pain in a dose-dependent manner. Compared to saline control, both 500 ng and 250 ng anti-IL-33 antibody increased the PWT of injured hindpaw in SNI rats, and reached the peak at 25 min post-injection. However, 100 ng of anti-IL-33 antibody had no obvious effect on the PWT of SNI rats.

In order to confirm the action of red nucleus IL-33 in pain modulation, the effect of exogenous IL-33 on the PWT of naive rats was observed after injected into the RN. As illustrated in Fig. 2F, intrarubral administration of various dosages of exogenous IL-33 (5.0 ng, 10 ng, 20 ng) to naive rats lowered the PWT of contralateral hindpaw and produced an obvious mechanical hypersensitivity in a dose-dependent manner, but had no influence on the PWT of ipsilateral hindpaw. Compared to saline control, both 20 ng and 10 ng IL-33 decreased the PWT of contralateral hindpaw in naive rats, and reached the maximum effect at 105 min post-injection. However, 5.0 ng IL-33 did not change the PWT of rats. Further studies showed that 20 ng of IL-33 injected into the RN was similar to the amount of IL-33 expressed in the RN of SNI rats (1 week post-injury) (Suppl. Fig. S1C). During the whole experiment process, no abnormal locomotion was observed in the rats after intrarubral application of anti-IL-33 antibody or IL-33 (Suppl. Fig. S1E). These results show that red nucleus IL-33 attends the early development of mononeuropathic pain and exerts an algesic effect.

### Red nucleus IL-33 facilitates the early development of mononeuropathic pain by activating NF-κB signaling pathway

**Fig. 3:**
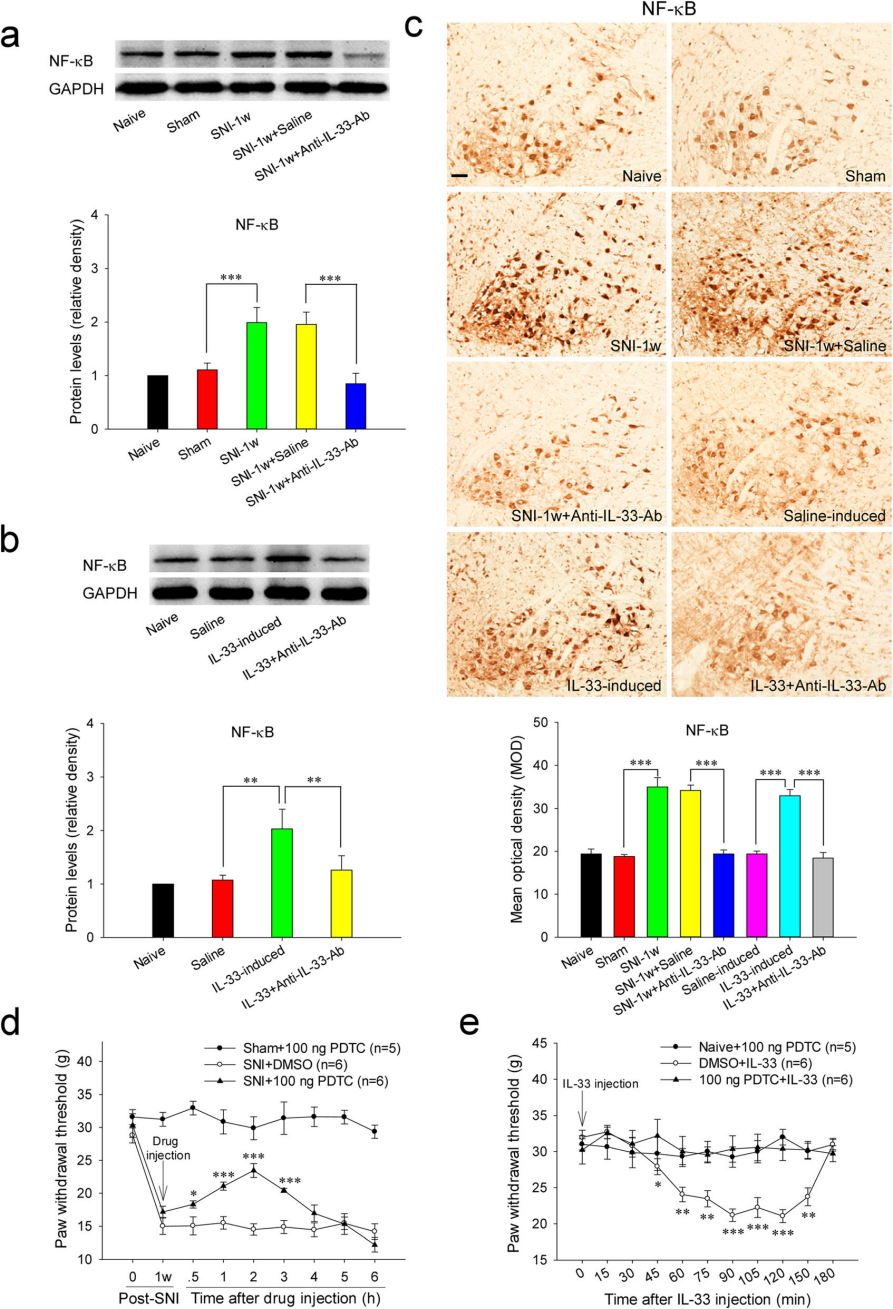
Red nucleus IL-33 facilitates the early development of mononeuropathic pain by activating NF-κB signaling pathway. **A** Western blotting showed an upregulated NF-κB in the RN at 1 week post-SNI, intrarubral administration of anti-IL-33 antibody restrained the overexpression of NF-κB (*n* = 6 per group, *F* = 12.766, *P* < 0.001). **B** Western blotting showed that intrarubral injection of IL-33 stimulated the protein expression of NF-κB in naive rats (*n* = 6 per group, *F* = 7.497, *P* = 0.003). **C** Immunohistochemistry demonstrated that NF-κB was increased in the RN of SNI rats (*F* = 41.250, *P* < 0.001) and IL-33-induced hypersensitivity rats (*F* = 34.509, *P* < 0.001) (*n* = 4 per group). **D** Intrarubral injection of NF-κB inhibitor PDTC at 1 week post-injury attenuated SNI-induced mononeuropathic pain compared to DMSO control (*n* = 5–6 per group, *F* = 135.298, *P* < 0.001). **E** PDTC pre-injected into the RN, 30 min ahead of IL-33 administration, relieved IL-33-evoked mechanical hypersensitivity compared to DMSO control (*n* = 5-6 per group, *F* = 97.341, *P* < 0.001). *P < 0.05, **P < 0.01, and ***P < 0.001. Scale bars = 50 μm

Previous studies have reported that IL-33 generates diverse biological effects through activating a variety of downstream signaling pathways, such as NF-κB, MAPKs, phosphatidylinositide 3-kinase/protein kinase B (PI3K/AKT), and JAK2/STAT3 [25]. Therefore, we first investigated whether NF-κB attends red nucleus IL-33-mediated algesic effect. In accordance with the upregulations of IL-33 and ST2, an overexpressed NF-κB was also detected in the RN at 1 week post-SNI compared to that in sham-surgery rats. Intrarubral administration of anti-IL-33 antibody at 1 week post-SNI significantly restrained the overexpression of NF-κB, while injection of normal saline had no any influence on the protein level of NF-κB (Fig. 3A, C). Intrarubral injection of PDTC, an inhibitor of NF-κB, at 1 week post-SNI elevated the mechanical PWT of injured hindpaw and significantly attenuated SNI-induced mononeuropathic pain compared to DMSO control. However, intrarubral application of PDTC did not change the mechanical PWT of sham-surgery rats (Fig. 3D).

Further studies demonstrated that intrarubral injection of IL-33 stimulated the protein expression of NF-κB in naive rats, while normal saline or IL-33 pre-absorbed by anti-IL-33 antibody did not alter the protein level of NF-κB (Fig. 3B, C). PDTC pre-injected into the RN, 30 min ahead of IL-33 administration, significantly attenuated IL-33-evoked mechanical hypersensitivity compared to DMSO control, while intrarubral injection of PDTC alone had no influence on the mechanical PWT of naive rats (Fig. 3E). These results indicate that red nucleus IL-33 facilitates the early development of mononeuropathic pain by activating NF-κB signaling pathway.

### Red nucleus IL-33 facilitates the early development of mononeuropathic pain by activating MAPK signaling pathway

**Fig. 4:**
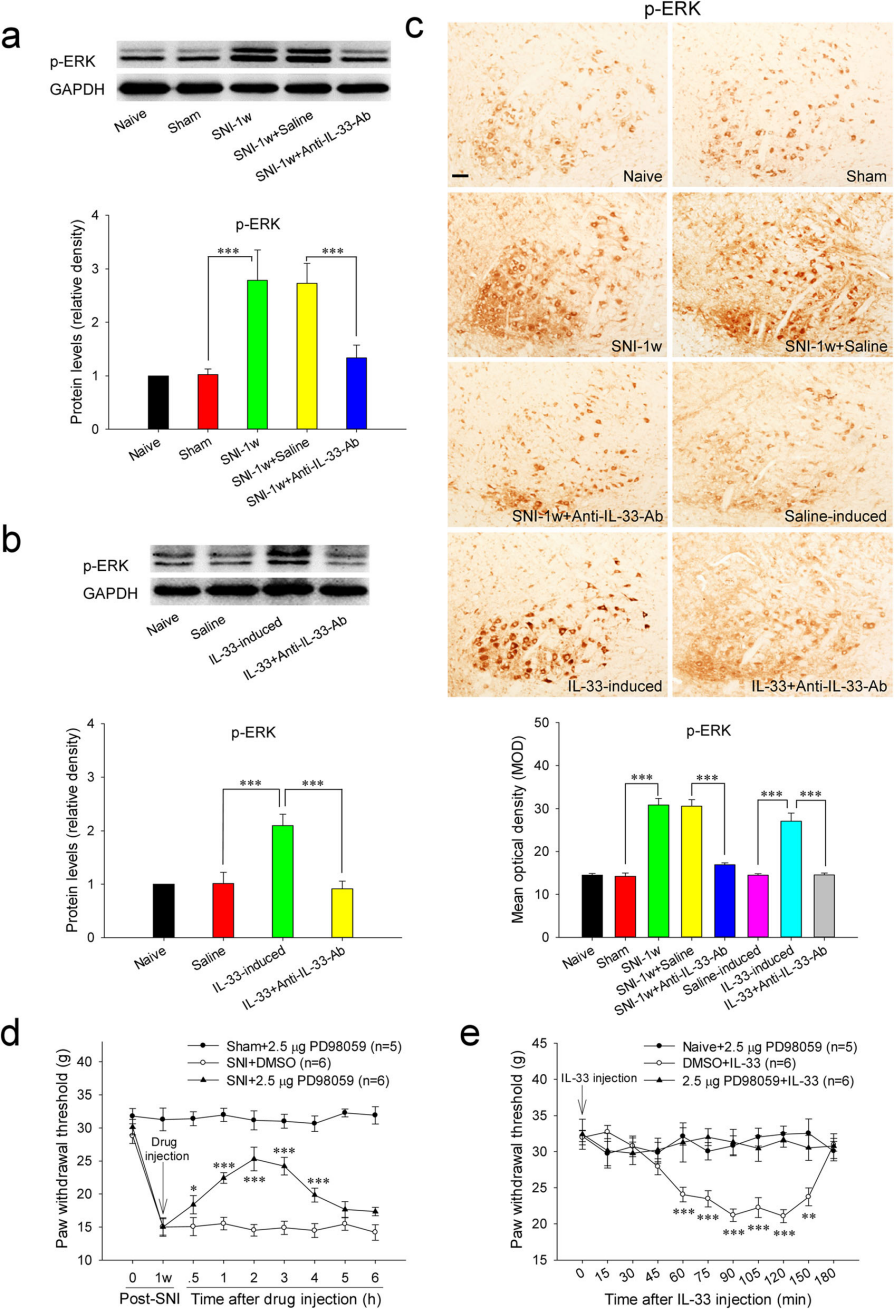
Red nucleus IL-33 facilitates the early development of mononeuropathic pain by activating ERK signaling pathway. **A** Western blotting showed that red nucleus p-ERK was increased at 1 week post-SNI, intrarubral injection of anti-IL-33 antibody inhibited the upregulation of p-ERK (*n* = 6 per group, *F* = 12.666, *P* < 0.001). **B** Western blotting indicated that intrarubral injection of IL-33 promoted the protein expression of p-ERK in naive rats (*n* = 6 per group, *F* = 14.495, *P* < 0.001). **C** Immunohistochemistry showed that p-ERK was upregulated in the RN of SNI rats (*F* = 69.719, *P* < 0.001) and IL-33-induced hypersensitivity rats (*F* = 40.034, *P* < 0.001) (*n* = 4 per group). **D** Intrarubral administration of ERK inhibitor PD98059 at 1 week post-injury attenuated SNI-induced mononeuropathic pain compared to DMSO control (*n* = 5-6 per group, *F* = 102.911, *P* < 0.001). **E** Intrarubral pre-injection of PD98059, 30 min before IL-33 administration, relieved IL-33-evoked mechanical hypersensitivity compared to DMSO control (*n* = 5–6 per group, *F* = 35.306, *P* = 0.002). *P < 0.05, **P < 0.01, and ***P < 0.001. *Scale bars =* 50 μm

**Fig. 5:**
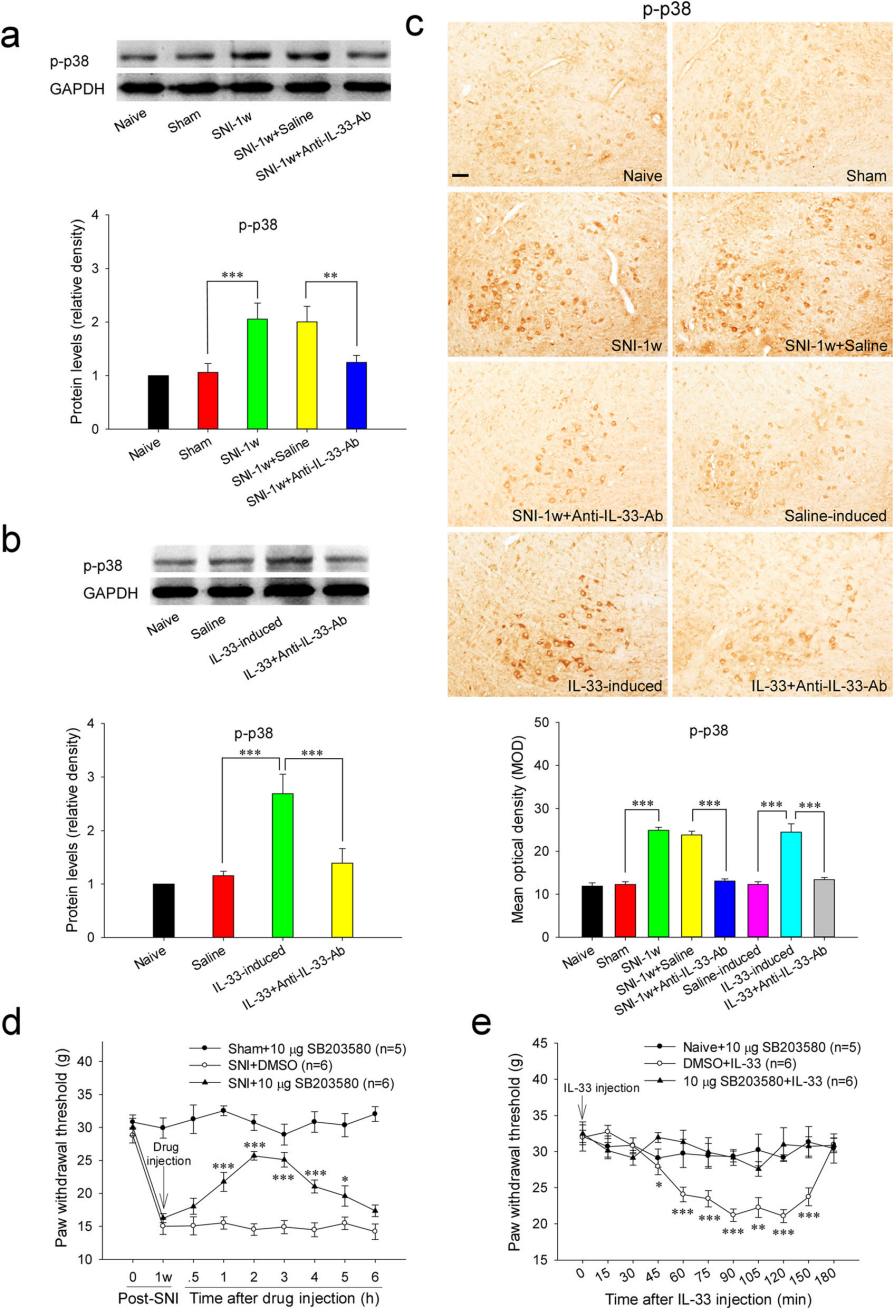
Red nucleus IL-33 facilitates the early development of mononeuropathic pain by activating p38 MAPK signaling pathway. **A** Western blotting showed an upregulated p-p38 MAPK in the RN at 1 week post-SNI, intrarubral administration of anti-IL-33 antibody restrained the increase of p-p38 MAPK (*n* = 6 per group, *F* = 13.002, *P* < 0.001). **B** Western blotting demonstrated that intrarubral injection of IL-33 stimulated the protein expression of p-p38 MAPK in naive rats (*n* = 6 per group, *F* = 18.974, *P* < 0.001). **C** Immunohistochemistry showed that p-p38 MAPK was increased in the RN of SNI rats (*F* = 88.711, *P* < 0.001) and IL-33-induced hypersensitivity rats (*F* = 27.208, *P* < 0.001) (*n* = 4 per group). **D** Intrarubral administration of p38 MAPK inhibitor SB203580 at 1 week post-injury attenuated SNI-induced mononeuropathic pain compared to DMSO control (*n* = 5–6 per group, *F* = 51.310, *P* < 0.001). **E** SB203580 pre-injected into the RN, 30 min ahead of IL-33 administration, relieved IL-33-evoked mechanical hypersensitivity compared to DMSO control (*n* = 5-6 per group, *F* = 39.381, *P* < 0.001). *P < 0.05, **P < 0.01, and ***P < 0.001. Scale bars = 50 μm

We next explored whether MAPK signaling pathways, including ERK, p38 MAPK, and JNK, contribute to red nucleus IL-33-mediated algesic effect. At 1 week post-SNI, the protein expressions of red nucleus p-ERK and p-p38 MAPK were significantly higher than that in sham-surgery rats. Intrarubral administration of anti-IL-33 antibody at 1 week post-SNI could suppress the overexpression of p-ERK and p-p38 MAPK (Fig. 4A, C and Fig. 5A, C). Whereas, the protein level of red nucleus p-JNK was not changed in SNI rats and not affected by administration of anti-IL-33 antibody (Suppl. Fig. S2). Intrarubral injection of normal saline had no influence on the expressions of p-ERK, p-p38 MAPK, and p-JNK in SNI rats. After administration of ERK inhibitor PD98059 or p38 MAPK inhibitor SB203580 to the RN at 1 week post-SNI, the PWT of the injured hindpaw was raised, and the mononeuropathic pain was significantly relieved. Intrarubral injection of PD98059 or SB203580 did not alter the mechanical PWT of sham-surgery rats (Fig. 4D and Fig. 5D).

Furthermore, intrarubral injection of IL-33 significantly promoted the protein expressions of p-ERK and p-p38 MAPK in naive rats, but did not alter the protein level of p-JNK compared to saline group. Neither normal saline nor IL-33 pre-absorbed by anti-IL-33 antibody affected the protein expressions of p-ERK, p-p38 MAPK, and p-JNK (Fig. 4B, C; Fig. 5B, C; and Suppl. Fig. S2). Intrarubral pre-injection of PD98059 or SB203580, 30 min before IL-33 administration, significantly attenuated IL-33-evoked mechanical hypersensitivity, while intrarubral injection of PD98059 or SB203580 alone had no influence on the mechanical PWT of naive rats (Fig. 4E and Fig. 5E). These results demonstrate that red nucleus IL-33 facilitates the early development of mononeuropathic pain by activating ERK and p38 MAPK in MAPK signaling pathway rather than JNK.

### Red nucleus IL-33 facilitates the early development of mononeuropathic pain by activating JAK2/STAT3 signaling pathway

**Fig. 6:**
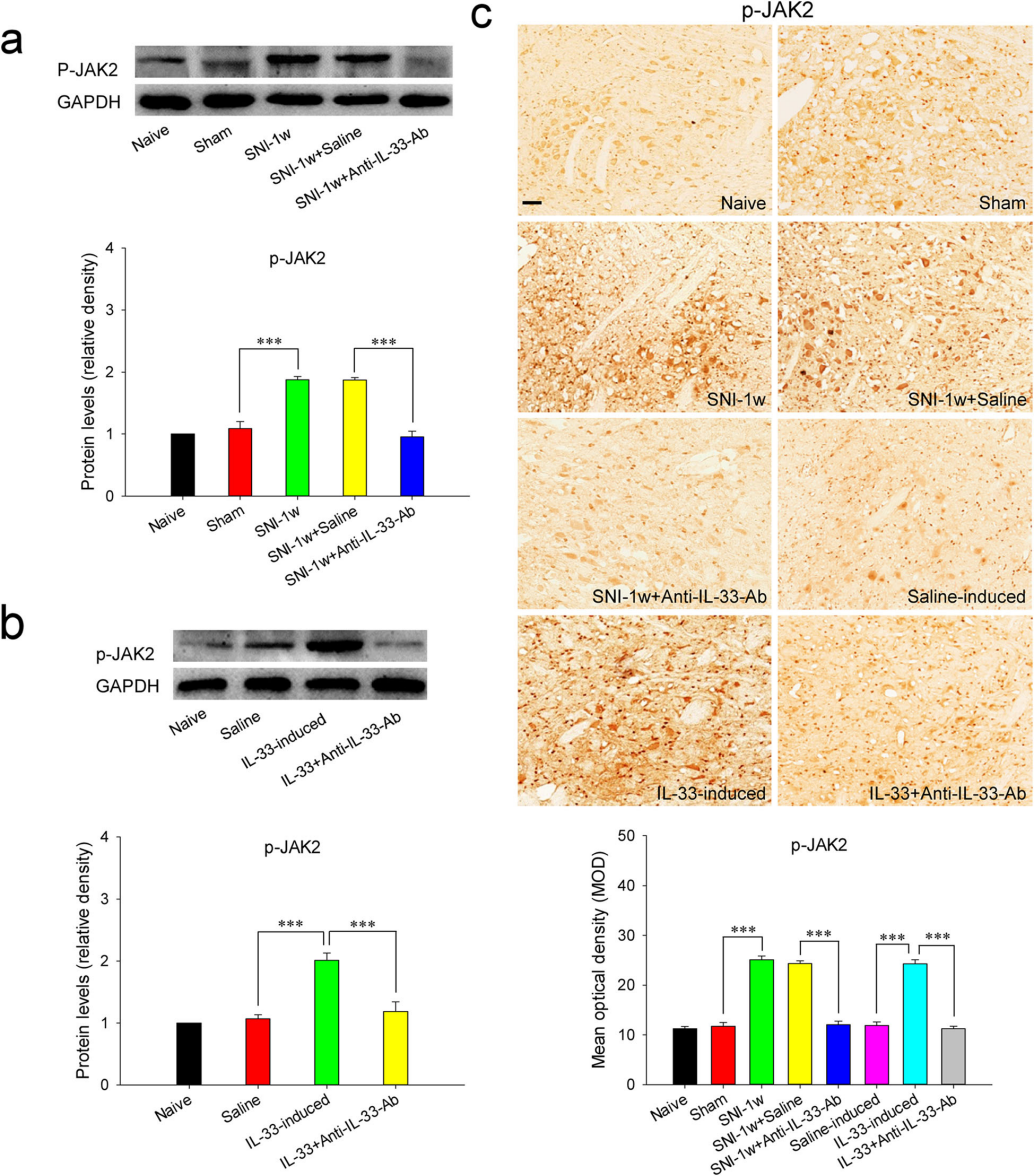
Red nucleus IL-33 facilitates the early development of mononeuropathic pain by activating JAK2/STAT3 signaling pathway. **A** Western blotting indicated that red nucleus p-JAK2 was upregulated at 1 week post-SNI, intrarubral injection of anti-IL-33 antibody inhibited the upregulation of p-JAK2 (*n* = 6 per group, *F* = 70.984, *P* < 0.001). **B** Western blotting showed that intrarubral administration of IL-33 promoted the protein expression of p-JAK2 in naive rats (*n* = 6 per group, *F* = 18.910, *P* < 0.001). **C** Immunohistochemistry showed that p-JAK2 was increased in the RN of SNI rats (*F* = 126.070, *P* < 0.001) and IL-33-induced hypersensitivity rats (*F* = 102.395, *P* < 0.001) (*n* = 4 per group). ***P < 0.001. Scale bars = 50 μm

**Fig. 7:**
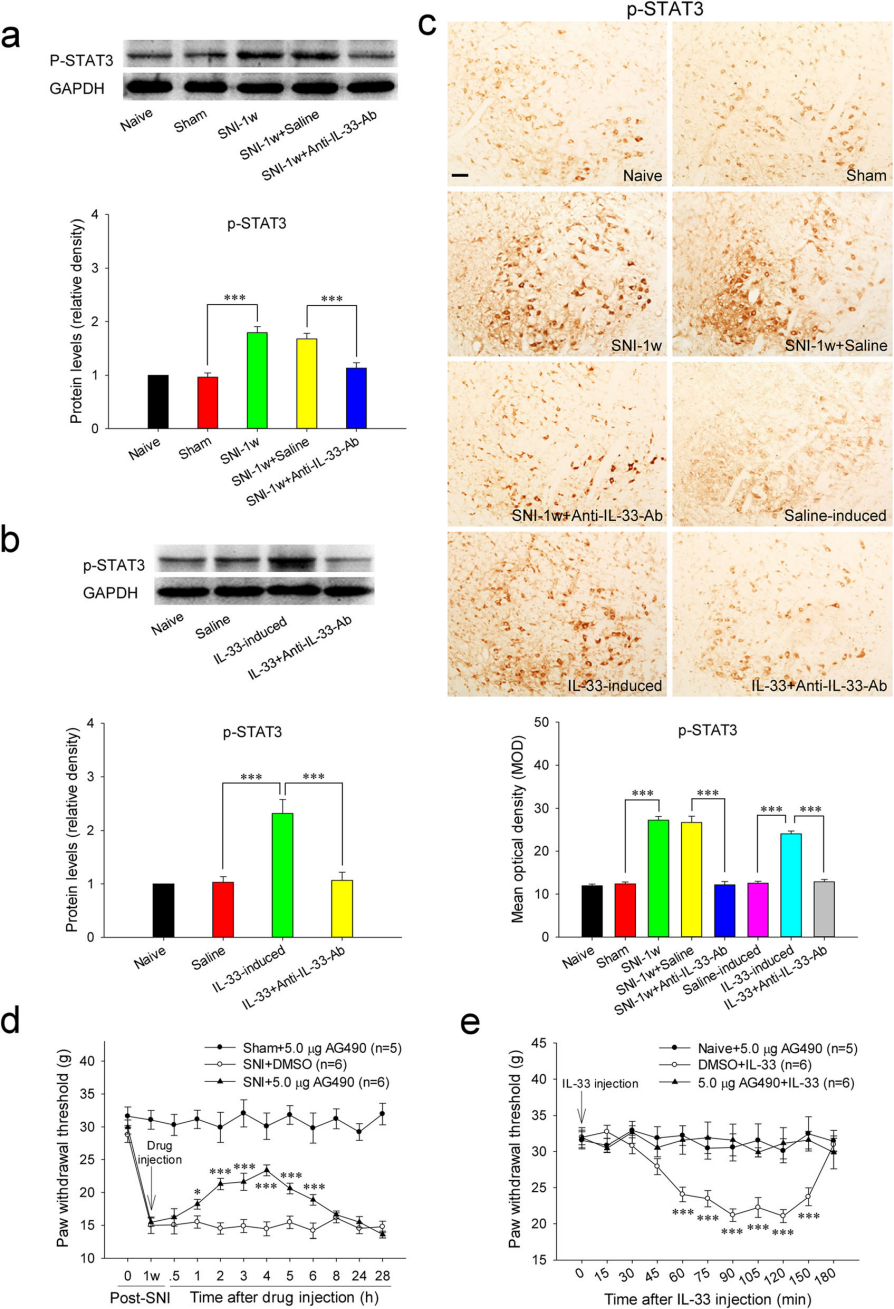
Red nucleus IL-33 facilitates the early development of mononeuropathic pain by activating JAK2/STAT3 signaling pathway. **A** Western blotting showed an upregulated p-STAT3 in the RN at 1 week post-SNI, intrarubral administration of anti-IL-33 antibody restrained the increase of p-STAT3 (*n* = 6 per group, *F* = 21.807, *P* < 0.001). **B** Western blotting demonstrated that intrarubral injection of IL-33 stimulated the protein expression of p-STAT3 in naive rats (*n* = 6 per group, *F* = 28.843, *P* < 0.001). **C** Immunohistochemistry showed that p-STAT3 was increased in the RN of SNI rats (*F* = 82.451, *P* < 0.001) and IL-33-induced hypersensitivity rats (*F* = 135.468, *P* < 0.001) (*n* = 4 per group). **D** Intrarubral administration of JAK2 inhibitor AG490 at 1 week post-injury attenuated SNI-induced mononeuropathic pain compared to DMSO control (*n* = 5-6 per group, *F* = 32.046, *P =* 0.002). **E** Intrarubral pre-injection of AG490, 30 min before IL-33 administration, relieved IL-33-evoked mechanical hypersensitivity compared to DMSO control (*n* = 5–6 per group, *F* = 73.740, *P* < 0.001). *P < 0.05 and ***P < 0.001. Scale bars = 50 μm

We then studied the action of JAK2/STAT3 signaling pathway in red nucleus IL-33-mediated algesic effect. At 1 week post-SNI, the protein expressions of red nucleus p-JAK2 and p-STAT3 were significantly upregulated compared to that in sham-surgery rats. Intrarubral administration of anti-IL-33 antibody at 1 week post-SNI significantly restrained the overproductions of p-JAK2 and p-STAT3, while injection of normal saline did not alter the protein levels of p-JAK2 and p-STAT3 (Fig. 6A, C and Fig. 7A, C). Administration of AG490, an inhibitor against JAK2, to the RN at 1 week post-SNI could elevate the mechanical PWT of injured hindpaw and relieve SNI-induced mononeuropathic pain, while intrarubral injection of AG490 had no effect on the mechanical PWT of sham-surgery rats (Fig. 7D).

Moreover, intrarubral administration of IL-33 significantly increased the protein expressions of p-JAK2 and p-STAT3 in naive rats, while application of normal saline or IL-33 pre-absorbed by anti-IL-33 antibody had no influence on p-JAK2 and p-STAT3 expressions (Fig. 6B, C and Fig. 7B, C). Intrarubral pre-injection of AG490, 30 min ahead of IL-33 administration, significantly attenuated IL-33-evoked mechanical hypersensitivity. However, intrarubral injection of AG490 alone did not change the mechanical PWT of naive rats (Fig. 7E). These results show that red nucleus IL-33 facilitates the early development of mononeuropathic pain by activating JAK2/STAT3 signaling pathway.

### PI3K/AKT signaling pathway does not attend red nucleus IL-33-mediated pain facilitation

In addition, we investigated whether PI3K/AKT signaling pathway has a hand in red nucleus IL-33-mediated algesic effect. As illustrated in Suppl. Fig. S3, our study results did not show obvious expression change of red nucleus p-AKT at 1 week post-SNI, and no significant difference was observed compared to sham-surgery rats. Intrarubral administration of anti-IL-33 antibody or normal saline at 1 week post-SNI had no impact on the protein level of p-AKT. In addition, intrarubral injection of IL-33 did not alter the expression of p-AKT in naive rats. These results display that PI3K/AKT signaling pathway does not attend red nucleus IL-33-mediated pain facilitation.

### Red nucleus IL-33 facilitates the early development of mononeuropathic pain by inducing TNF-α through ERK, p38 MAPK, and JAK2/STAT3 signaling pathways

**Fig. 8:**
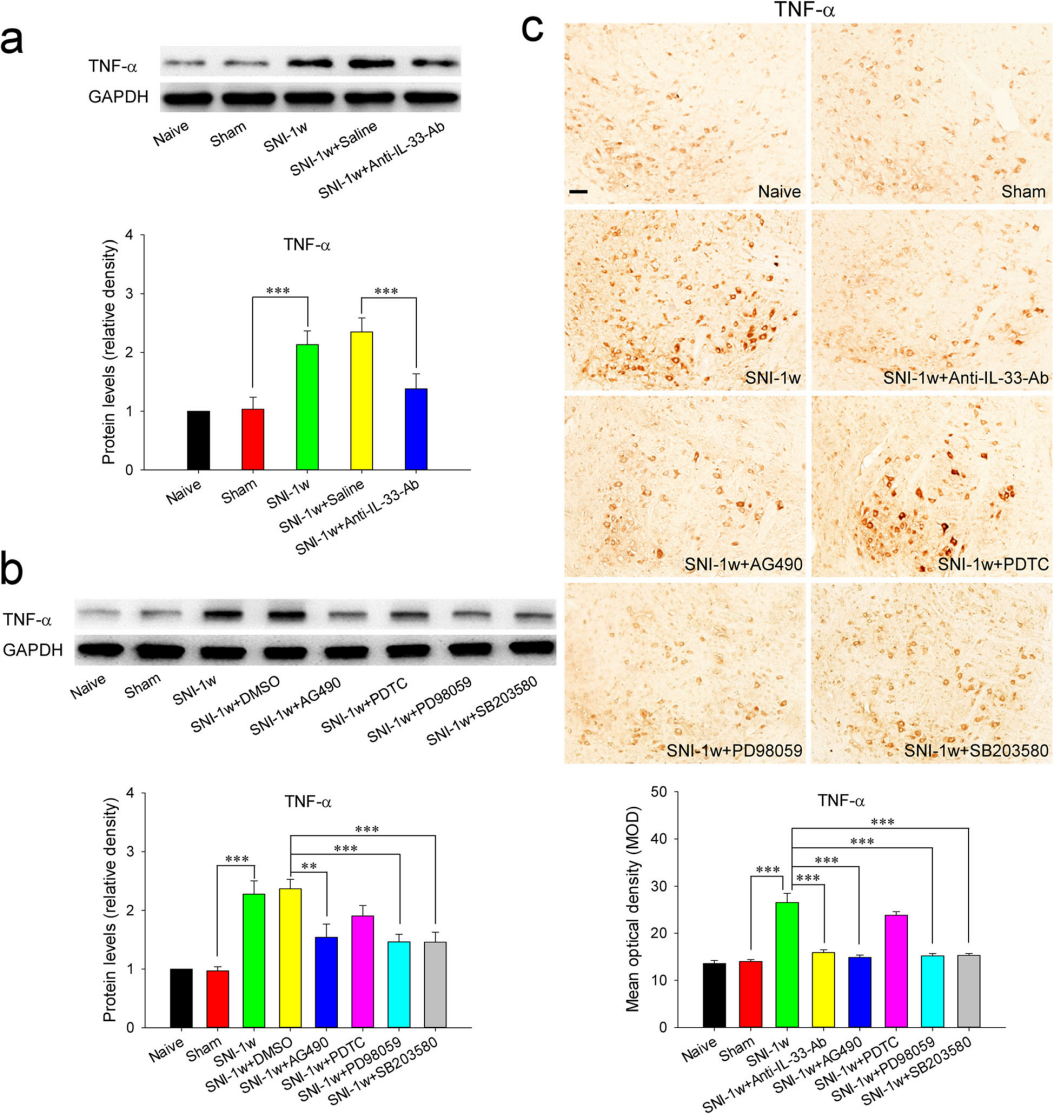
Red nucleus IL-33 facilitates the early development of mononeuropathic pain by inducing TNF-α through activating ERK, p38 MAPK and JAK2/STAT3 signaling pathways. **A** Western blotting showed that red nucleus TNF-α was increased at 1 week post-SNI, intrarubral injection of anti-IL-33 antibody at 1 week post-SNI suppressed the overexpression of TNF-α (*n* = 6 per group, *F* = 14.302, *P* < 0.001). **B** Western blotting displayed that intrarubral administration of PD98059, SB203580, or AG490 at 1 week post-SNI inhibited the production of TNF-α (*n* = 6 per group, *F* = 13.157, *P* < 0.001). **C** Immunohistochemistry showed that red nucleus TNF-α was upregulated at 1 week post-SNI, intrarubral injection of anti-IL-33 antibody, PD98059, SB203580, or AG490 at 1 week post-SNI suppressed the production of TNF-α (*n* = 4 per group, *F* = 33.029, *P* < 0.001). **P < 0.01 and ***P < 0.001. Scale bars = 50 μm

**Fig. 9:**
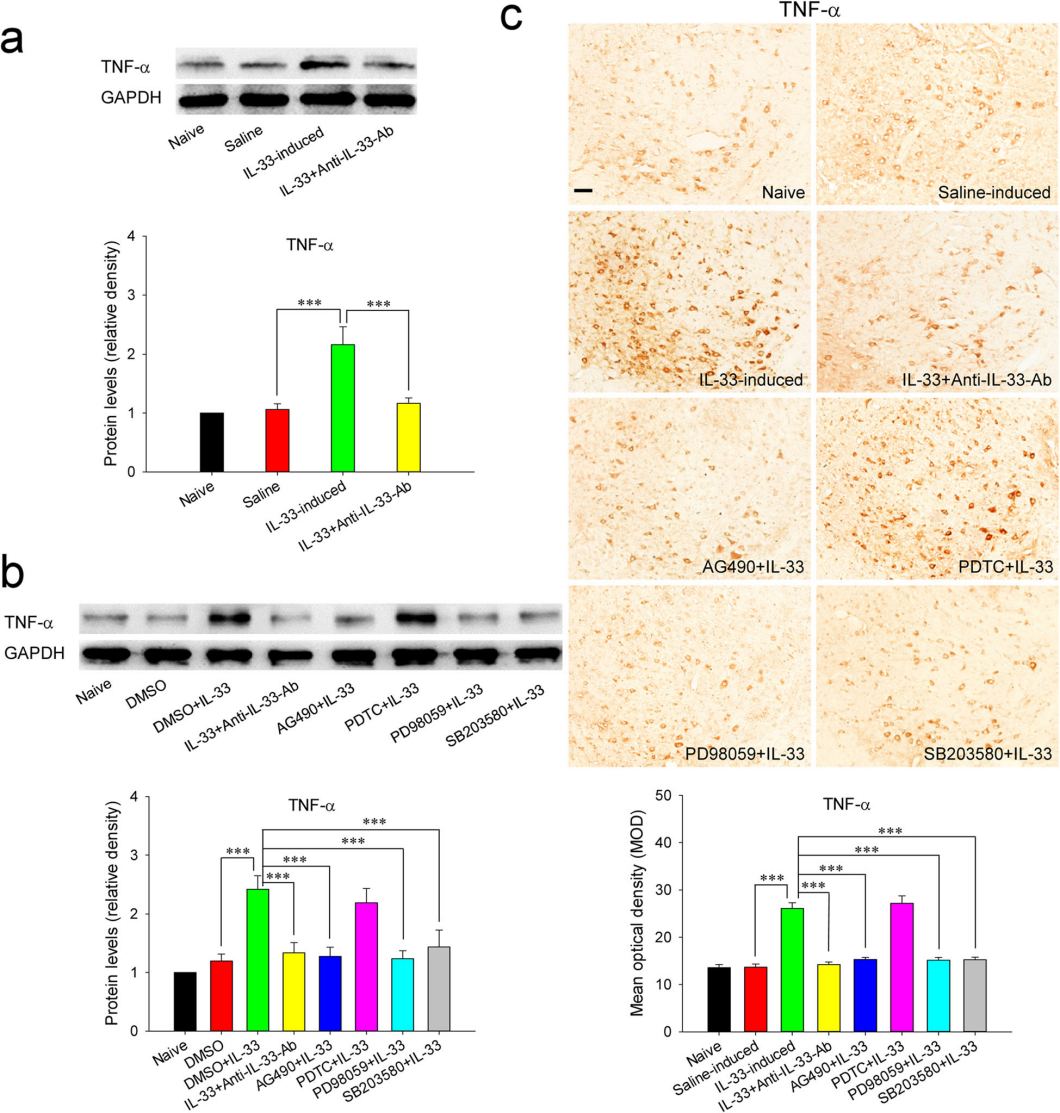
Red nucleus IL-33 evokes mechanical hypersensitivity by inducing TNF-α through activating ERK, p38 MAPK, and JAK2/STAT3 signaling pathways. **A** Western blotting indicated that intrarubral administration of IL-33 stimulated the secretion of TNF-α in naive rats (*n* = 6 per group, *F* = 15.143, *P* < 0.001). **B** Western blotting showed that intrarubral pre-injection of PD98059, SB203580, or AG490, 30 min before IL-33 administration, restrained IL-33-induced overexpression of TNF-α in naive rats (*n* = 6 per group, *F* = 9.812, *P* < 0.001). **C** Immunohistochemistry showed that intrarubral injection of IL-33 potentiated the secretion of TNF-α in naive rats, intrarubral pre-injection of PD98059, SB203580, or AG490, 30 min prior to IL-33 administration, inhibited IL-33-induced overexpression of TNF-α in naive rats (*n* = 4 per group, *F* = 44.310, *P* < 0.001). *** *P* < 0.001. Scale bars = 50 μm

Our previous studies have identified that red nucleus TNF-α is increased at 1 week post-SNI [18], and exerts an algesic effect in pain regulation [16]. Thus, we further explored whether red nucleus IL-33 generates algesic effect by inducing the production of TNF-α. The results showed that intrarubral injection of anti-IL-33 antibody at 1 week post-SNI significantly suppressed the overexpression of TNF-α, while administration of normal saline had no any influence on the protein level of TNF-α (Fig. 8A, C). Moreover, intrarubral injection of exogenous IL-33 significantly potentiated the secretion of TNF-α in naive rats, but administration of normal saline or IL-33 pre-absorbed by anti-IL-33 antibody did not affect the expression of TNF-α (Fig. 9A, C). These results indicate that red nucleus IL-33 facilitates the early development of mononeuropathic pain by inducing the production of TNF-α.

Based on the above findings, we next studied the signaling transduction pathways through which IL-33 stimulates the production of TNF-α. Our study results showed that intrarubral administration of PD98059, SB203580, or AG490 at 1 week post-SNI significantly inhibited the expression of TNF-α, but PDTC and DMSO did not affect the protein level of TNF-α (Fig. 8B, C). Intrarubral pre-injection of PD98059, SB203580, or AG490, 30 min before IL-33 administration, significantly restrained IL-33-induced overexpression of TNF-α in naive rats, whereas pre-injection of PDTC and DMSO had no obvious influence on IL-33-induced production of TNF-α (Fig. 9B, C). These results display that red nucleus IL-33 facilitates the early development of mononeuropathic pain by inducing TNF-α through activating ERK, p38 MAPK, and JAK2/STAT3, but not NF-κB, signaling pathways.

## Discussion

IL-33 is a newly discovered cytokine that has been implicated in the physiological and pathological processes of nervous system. Recently, a number of studies have proven that IL-33 contributes to the development of pathological pain [37–42]. However, the effect of supraspinal IL-33 in pain modulation is largely unknown so far. In the current study, we have investigated the action of red nucleus IL-33/ST2 in the early development of mononeuropathic pain in male rats since the mechanism of mononeuropathic pain is sex-dependent [32]. Following SNI, an obvious mechanical hypersensitivity is induced in rats at 3 days and aggravated at 1 week later. Consistent with the behavioral alterations, a short-term elevation of IL-33 is detected in the RN, starting at 3 days and returning to the basic level at 2 weeks post-SNI, implying that red nucleus IL-33 is involved mainly in the early development, but not in the late maintenance of mononeuropathic pain. Interestingly, red nucleus ST2 is also increased at 3 days post-SNI but still kept at a high level 3 weeks later. Thus, we speculate that the difference of decline time between IL-33 and ST2 may be related to their different upstream regulatory molecules. These results are parallel to previous findings that spinal IL-33 and ST2 are markedly increased at 3 days post-nerve injury [30, 39, 40]. However, several studies have provided inconsistent results that spinal IL-33 and ST2 are upregulated at 7 days or 14 days post-nerve injury [41, 42]. To further explore the effect of red nucleus IL-33 in the early development of mononeuropathic pain, we have detected the behavioral alterations in SNI rats after blocking IL-33 signaling. Our data show that intrarubral administration of anti-IL-33 antibody at 1 week post-SNI attenuates mechanical hypersensitivity of rats, indicating that red nucleus IL-33 facilitates the early development of mononeuropathic pain. Additionally, exogenous IL-33 injected into the RN of naive rats can dose-dependently evoke mechanical hypersensitivity, which further confirms the algesic effect of red nucleus IL-33. However, it is noteworthy that mechanical hypersensitivity evoked by 20 ng of IL-33 in naive rats is weaker than that induced by SNI at 1 week, although this dose is similar to the amount of IL-33 expressed in the RN of SNI rats, implying some molecules excepting IL-33 may be also involved in the early development of mononeuropathic pain. Our findings are in accord with previously reported that administration of exogenous IL-33 to normal animals induces pain hypersensitivity, while blocking IL-33/ST2 signaling inhibits pathological pain [39, 43, 44].

Previous studies and the data from single cell RNA sequencing databases have shown that both mRNA and protein of IL-33 are detected in astrocytes [39, 45–49], oligodendrocytes [39, 48–50], neurons [39, 49], microglia [39, 49, 50], and endothelial cells [45, 46, 49] in various human and animal tissues. Nevertheless, the expression levels of IL-33 across spinal cord and brain regions are not uniform and constant, which potentially reflects specific actions of IL-33 in region-related neuronal activities and disorders. Here, we demonstrate that red nucleus IL-33 is produced mainly by oligodendrocytes, endothelial cells, and a small amount by neurons, astrocytes, and microglia in naive rats. Following SNI, IL-33 is elevated in RN neurons, oligodendrocytes, and microglia but not astrocytes and endothelial cells. Our findings suggest that RN neurons, oligodendrocytes, and microglia contribute to the early development of mononeuropathic pain through upregulating the expression of IL-33. Similar to the expression of IL-33, the distribution of ST2 is also inconsistent in different regions of center nerve system. Some studies show that ST2 is expressed mainly in microglia [45, 48, 49], whereas others demonstrate its expression in astrocytes [40, 45, 48], neurons [40, 48], oligodendrocytes [40, 48], and endothelial cells [48, 51]. Our data indicate that red nucleus ST2 is mainly expressed in oligodendrocytes, microglia, endothelial cells, and a minor expression in neurons and astrocytes in naive rats. It is also increased in neurons, oligodendrocytes, and microglia after SNI. These results display that red nucleus IL-33 perhaps mediates the early development of mononeuropathic pain by activating neurons, oligodendrocytes, and microglia through autocrine and/or paracrine manners. ST2 expressed in RN astrocytes and endothelial cells do not show obvious changes, implying that these cells may be not the direct responders for IL-33 mediating pain regulation.

Further studies have discovered that IL-33 contributes to pathological pain through NF-κB, ERK, p38 MAPK, JNK, JAK2/STAT3, and/or PI3K/AKT signaling pathways [30, 39, 40]. However, it remains unclear whether these signaling pathways participate in red nucleus IL-33-mediated algesic effect. Thus, we have detected the expression changes of these signaling molecules in the RN of SNI rats and their roles in the early development of mononeuropathic pain. Parallel to the upregulations of IL-33 and ST2, red nucleus NF-κB, p-ERK, p-p38 MAPK, and p-JAK2/p-STAT3, but not p-JNK and p-AKT, are elevated at 1 week post-SNI. Blockade of NF-κB, ERK, p38 MAPK, or JAK2/STAT3 with corresponding inhibitor relieves SNI-induced mechanical hypersensitivity. These results suggest an algesic effect of red nucleus NF-κB, ERK, p38 MAPK, and JAK2/STAT3, but not JNK and PI3K/AKT, in the early development of mononeuropathic pain. Then, we have further addressed whether the activation of NF-κB, ERK, p38 MAPK, and JAK2/STAT3 are caused by IL-33. Our data show that blocking red nucleus IL-33 with anti-IL-33 antibody at 1 week post-SNI inhibits the overexpression of NF-κB, p-ERK, p-p38 MAPK, and p-JAK2/p-STAT3, suggesting that red nucleus IL-33 facilitates the early development of mononeuropathic pain through activating NF-κB, ERK, p38 MAPK, and JAK2/STAT3 signaling pathways. This conclusion is further proven by the experiments performed in naive rats, that is, intrarubral administration of IL-33 in naive rats potentiates the productions of NF-κB, p-ERK, p-p38 MAPK, and p-JAK2/p-STAT3, but has no effect on the expressions of p-JNK and p-AKT. Pre-injection of inhibitor against NF-κB, ERK, p38 MAPK, or JAK2/STAT3 attenuates IL-33-evoked mechanical hypersensitivity. Previous studies have reported that ERK and JNK located in astrocytes and neurons, p38 MAPK located in microglia, and JAK2/STAT3 located in astrocytes are activated during the development of pathological pain [30, 52, 53]. Nevertheless, future studies are required to address the cellular localization and interaction of NF-κB, ERK, p38 MAPK, and JAK2/STAT3 signaling pathways in the RN of rats.

The latest studies have shown that IL-33 regulates the transcription of proinflammatory cytokines [44, 54]. It has been reported that IL-33 facilitates pathological pain by stimulating the secretion of TNF-α, IL-1β, and/or IL-6 [31, 38–40]. In the current study, red nucleus IL-33 begins to increase at 3 days post-SNI, reaches to peak at 1 week, and returns to normal level at 2 weeks. Combining with our previously reported that the expression of red nucleus TNF-α is increased at 1 week post-SNI [18], while IL-1β and IL-6 are increased at 2 or 3 weeks post-SNI [13, 17], it is reasonable to infer that TNF-α, but not IL-1β and IL-6, may be the direct downstream molecule of IL-33. Therefore, we have further studied whether red nucleus IL-33 mediates algesic effect by inducing TNF-α. Our data indicate that blocking red nucleus IL-33 at 1 week post-SNI can suppress the overexpression of TNF-α, while intrarubral administration of IL-33 in naive rats promotes the production of TNF-α. These results strongly suggest that red nucleus IL-33 exerts an algesic effect at least in part by stimulating TNF-α expression, although it may has other downstream molecules that we do not know yet. In the present study, we have proven that red nucleus IL-33 mediates algesic effect through activating NF-κB, ERK, p38 MAPK and JAK2/STAT3. However, which signaling pathways attend IL-33-induced production of TNF-α remains unclear. Further studies demonstrate that intrarubral administration of inhibitor of ERK, p38 MAPK, or JAK2/STAT3 (but not NF-κB) inhibits the overexpression of TNF-α in SNI rats and IL-33-induced hypersensitivity rats, displaying that red nucleus IL-33 induces the production of TNF-α through activating ERK, p38 MAPK, and JAK2/STAT3, but not NF-κB, signaling pathways. Although our data have clearly shown that NF-κB is also involved in IL-33-mediated algesic effect, the downstream molecules regulated by NF-κB in this process remain unclear and need further investigations.

## Conclusions

Taken together, our work provides evidence that red nucleus IL-33 contributes to the early development of mononeuropathic pain, and generates an algesic effect via activating NF-κB, ERK, p38 MAPK, and JAK2/STAT3 signaling pathways. Red nucleus IL-33 facilitates the early development of mononeuropathic pain at least in part by inducing TNF-α through activating ERK, p38 MAPK, and JAK2/STAT3 signaling pathways, and maybe a potential target for the treatment of mononeuropathic pain.

## Supplementary Information

**Additional file 1: Suppl. Fig. S1.** Histomorphological identification of RN, the analysis of IL-33 in the RN, the specificity verification of anti-IL-33 antibody and anti-ST2 antibody, and the measurement of locomotion. **A** 0.1% toluidine blue staining exhibited the injection site in the RN. **B** The fresh tissue of RN. **C** Western blotting showed that intrarubral injection of 20 ng IL-33 to naive rats could mimic the amount of IL-33 in the RN of SNI rats (1 week post-injury) (*n* = 4 per group). **D** No specific signals of IL-33 and ST2 (Red) were detected respectively in the RN of *IL-33*^-/-^ and *ST2*^-/-^ mice. **E** Footprint test showed that intrarubral application of anti-IL-33 antibody to SNI rats or IL-33 to naive rats did not affect the locomotion of animal (*n* = 6-9 per group). Abbreviations: Aq, aqueduct; PAG, periaqueductal gray; RN, red nucleus. Scale bars = 50 μm.

**Additional file 2: Suppl. Fig. S2.** JNK signaling pathway does not attend red nucleus IL-33-mediated pain facilitation. **A** Western blotting showed no expression alteration of p-JNK in the RN at 1 week post-SNI (*n* = 6 per group). **B** Western blotting indicated that intrarubral injection of IL-33 did not affect the protein level of p-JNK in naive rats (*n* = 6 per group). **C** Immunohistochemical staining demonstrated no expression changes of p-JNK in the RN of SNI rats and IL-33-induced hypersensitivity rats (*n* = 4 per group). Scale bars = 50 μm.

**Additional file 3: Suppl. Fig. S3.** PI3K/AKT signaling pathway does not attend red nucleus IL-33-mediated pain facilitation. **A** Western blotting showed no expression alteration of p-AKT in the RN at 1 week post-SNI (*n* = 6 per group). **B** Western blotting indicated that intrarubral injection of IL-33 did not alter the expression of p-AKT in naive rats (*n* = 6 per group). **C** Immunohistochemical staining demonstrated no expression changes of p-AKT in the RN of SNI rats and IL-33-induced hypersensitivity rats (*n* = 4 per group). Scale bars = 50 μm.

## Data Availability

The datasets used and/or analyzed during the current study are available from the corresponding author on reasonable request.

## Abbreviations

ANOVA: Analysis of variance
ERK: Extracellular signal-regulated kinase
GAPDH: Glyceraldehyde-3-phosphate dehydrogenase
IL: Interleukin
JAK2/STAT3: Janus kinase 2/signal transducer and activator of transcription 3
JNK: c-Jun N-terminal kinase
NF-κB: Nuclear factor-κB
p38 MAPK: p38 mitogen-activated protein kinase
PI3K/AKT: Phosphatidylinositide 3-kinase/protein kinase B
PWT: Paw withdrawal threshold
RN: Red nucleus
SNI: Spared nerve injury
TGF-β: Transforming growth factor-β
TNF-α: Tumor necrosis factor-α
