# Supplementary figures and images for: Red nucleus IL-33 facilitates the early development of mononeuropathic pain in male rats by inducing TNF-α through activating ERK, p38 MAPK, and JAK2/STAT3
Source: J Neuroinflammation. 2021 Jul 5;18:150. doi: 10.1186/s12974-021-02198-9 (PMC8258957; doi:10.1186/s12974-021-02198-9)

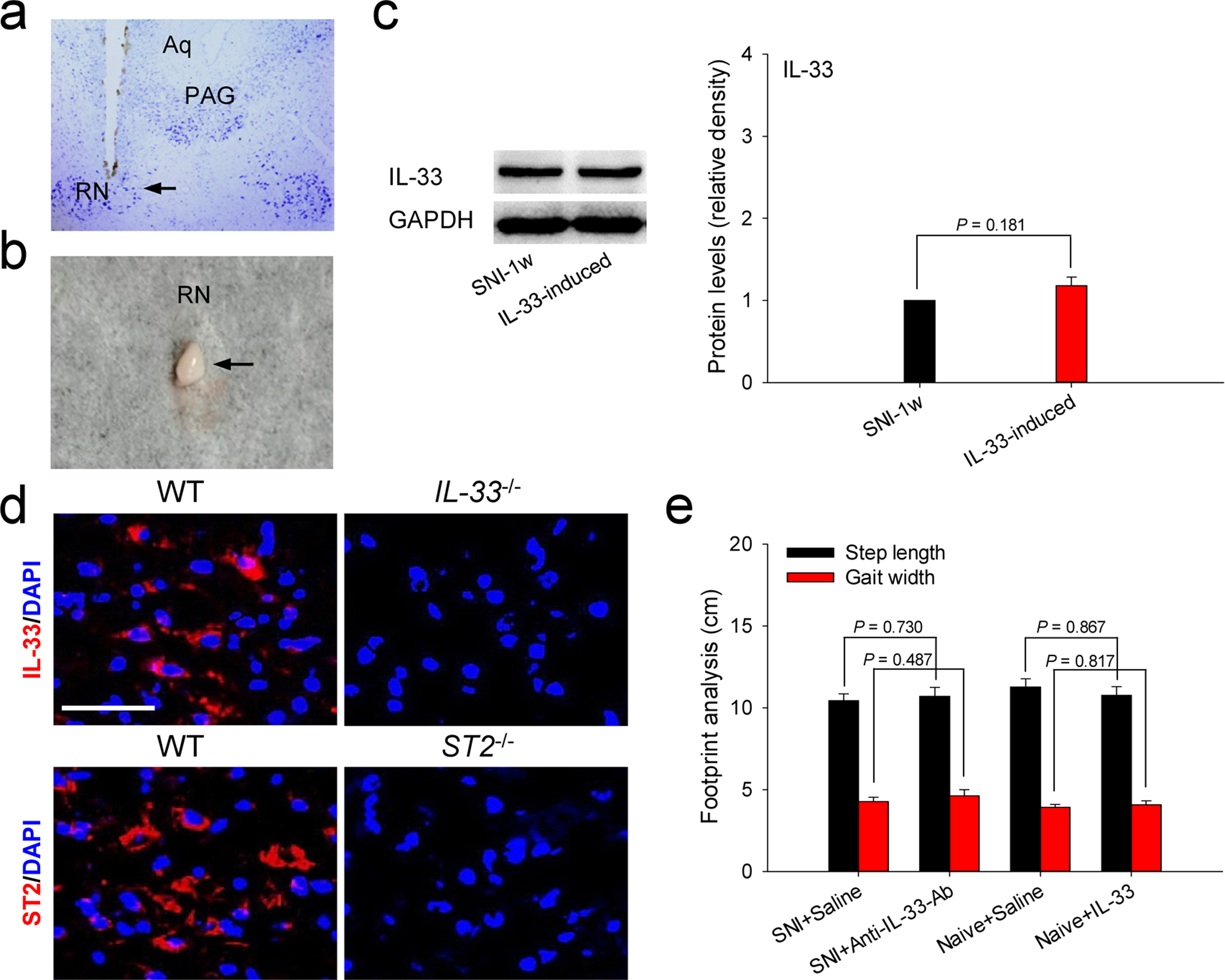

Supplement: Supplementary file 1 — Additional file 1: Suppl. Fig. S1. Histomorphological identification of RN, the analysis of IL-33 in the RN, the specificity verification of anti-IL-33 antibody and anti-ST2 antibody, and the measurement of locomotion. A 0.1% toluidine blue staining exhibited the injection site in the RN. B The fresh tissue of RN. C Western blotting showed that intrarubral injection of 20 ng IL-33 to naive rats could mimic the amount of IL-33 in the RN of SNI rats (1 week post-injury) (n = 4 per group). D No specific signals of IL-33 and ST2 (Red) were detected respectively in the RN of IL-33-/- and ST2-/- mice. E Footprint test showed that intrarubral application of anti-IL-33 antibody to SNI rats or IL-33 to naive rats did not affect the locomotion of animal (n = 6-9 per group). Abbreviations: Aq, aqueduct; PAG, periaqueductal gray; RN, red nucleus. Scale bars = 50 μm. [file 12974_2021_2198_MOESM1_ESM.tiff]

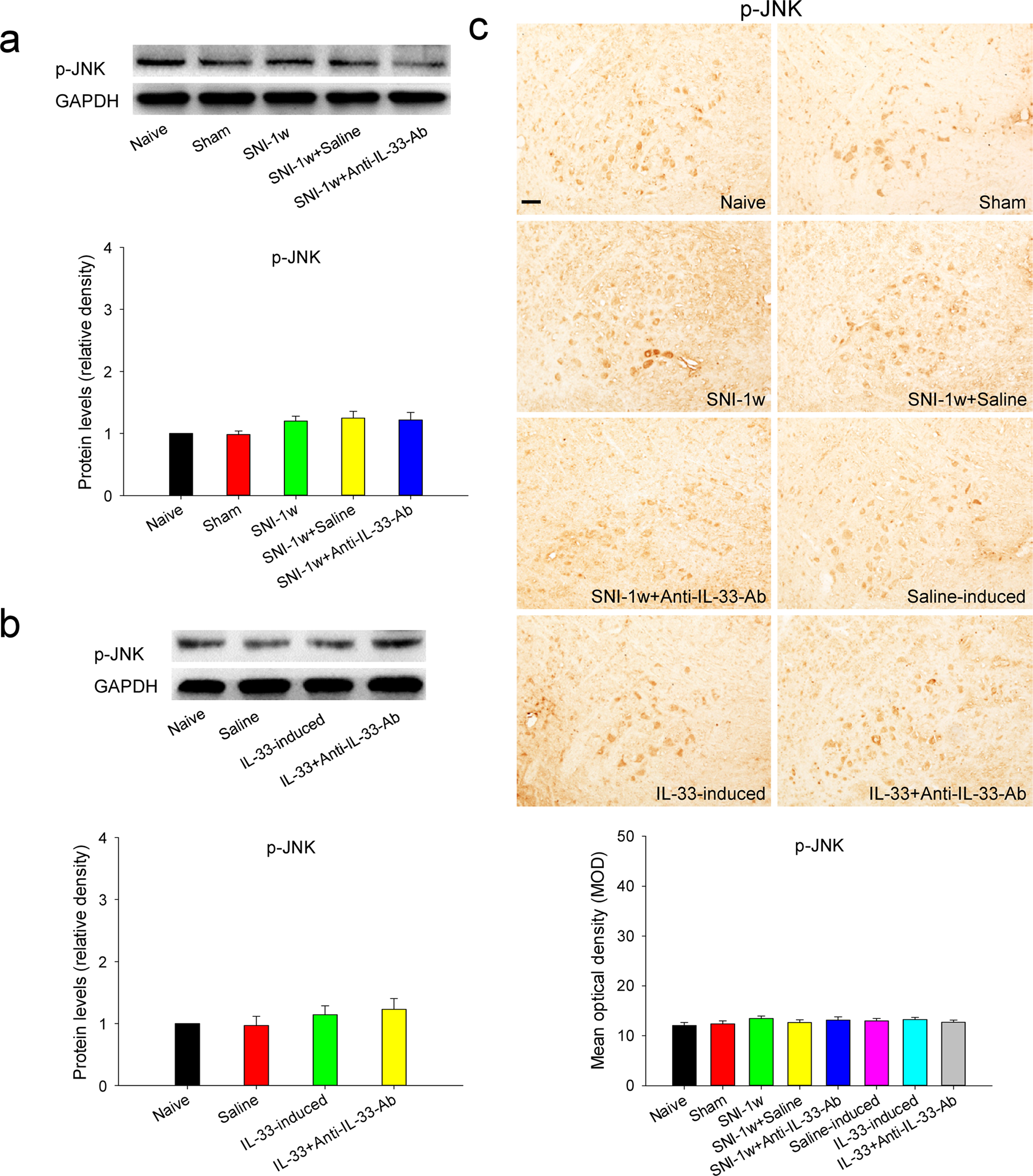

Supplement: Supplementary file 2 — Additional file 2: Suppl. Fig. S2. JNK signaling pathway does not attend red nucleus IL-33-mediated pain facilitation. A Western blotting showed no expression alteration of p-JNK in the RN at 1 week post-SNI (n = 6 per group). B Western blotting indicated that intrarubral injection of IL-33 did not affect the protein level of p-JNK in naive rats (n = 6 per group). C Immunohistochemical staining demonstrated no expression changes of p-JNK in the RN of SNI rats and IL-33-induced hypersensitivity rats (n = 4 per group). Scale bars = 50 μm. [file 12974_2021_2198_MOESM2_ESM.tiff]

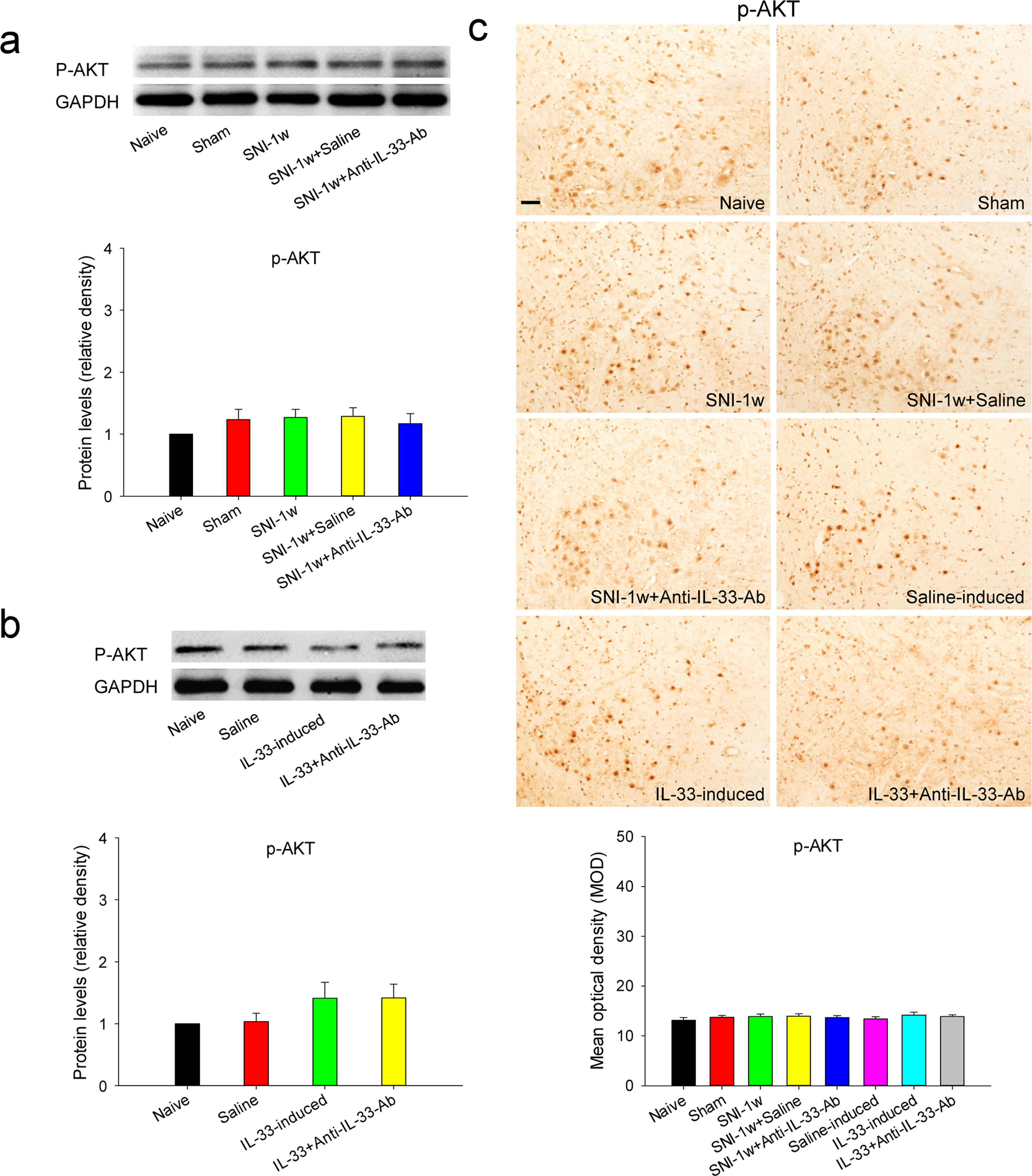

Supplement: Supplementary file 3 — Additional file 3: Suppl. Fig. S3. PI3K/AKT signaling pathway does not attend red nucleus IL-33-mediated pain facilitation. A Western blotting showed no expression alteration of p-AKT in the RN at 1 week post-SNI (n = 6 per group). B Western blotting indicated that intrarubral injection of IL-33 did not alter the expression of p-AKT in naive rats (n = 6 per group). C Immunohistochemical staining demonstrated no expression changes of p-AKT in the RN of SNI rats and IL-33-induced hypersensitivity rats (n = 4 per group). Scale bars = 50 μm. [file 12974_2021_2198_MOESM3_ESM.tiff]
